# Morningness/eveningness in gestational diabetes mellitus: clinical characteristics and maternal-neonatal outcomes

**DOI:** 10.20945/2359-3997000000515

**Published:** 2022-09-20

**Authors:** Cristina Figueiredo Sampaio Facanha, Victória Sudário Alencar, Paula Soares Machado, Rejane Belchior Lima Macêdo, Pedro Felipe Carvalhedo de Bruin, Adriana Costa e Forti, Thaine Mirla Rocha, Veralice Meireles Sales de Bruin

**Affiliations:** 1 Universidade Federal do Ceará Departamento de Medicina Fortaleza CE Brasil Departamento de Medicina, Universidade Federal do Ceará, Fortaleza, CE, Brasil; 2 Centro Universitário Christus Departamento de Medicina Fortaleza CE Brasil Departamento de Medicina, Centro Universitário Christus, Fortaleza, CE, Brasil; 3 Estadual de Saúde do Ceará Centro Integrado de Diabetes e Hipertensão do Ceará (CIDH) Fortaleza CE Brasil Centro Integrado de Diabetes e Hipertensão do Ceará (CIDH), Secretaria Estadual de Saúde do Ceará, Fortaleza, CE, Brasil

**Keywords:** Circadian rhythm, pregnancy outcome, diabetes, gestational, sleep wake disorders, actigraphy

## Abstract

**Objective:**

This study aims to evaluate the impact of morning-evening preference in pregnancy outcomes in gestational diabetes mellitus (GDM).

**Materials and methods:**

This is a prospective cohort study of 2nd-3rd trimester GDM outpatient care in Fortaleza, Brazil (2018-2020). Eveningness was defined by the Horne-Östberg Morningness-Eveningness-Questionnaire (MEQ ≤ 41). Furthermore, we obtained a 7-day actigraphic register. Subjective sleep quality, daytime somnolence, insomnia, fatigue and depressive symptoms were also evaluated. Associations with pregnancy outcomes were investigated.

**Results:**

Among 305 patients with GDM, evening preference was found in 21 (6.9%). Patients with evening preference had worse sleep quality (p < 0.01), greater severity of insomnia (p < 0.005), fatigue (p < 0.005) and depressive symptoms (<0.009). Evening chronotype was associated with preeclampsia [p = 0.01; OR = 0.27; CI 0.09-0.79] and a greater need for admission to a neonatal intensive care unit (NICU) [p = 0.02; OR = 0.23; CI .0.06-0.80]. A lower MEQ score confirmed an association with preeclampsia [p = 0.002; OR = 0.94; CI 0.90-0.97] and this was maintained after controlling for age, arterial hypertension, sleep quality, fatigue and depressive symptoms [p < 005; OR = 0.91; CI 0.87-0.95].

**Conclusion:**

In GDM, patients with evening preference had worse sleep quality, more insomnia, fatigue, and depressive symptoms. Furthermore, eveningness was independently associated with preeclampsia. These results indicate the important role of eveningness in adverse pregnancy outcomes.

## INTRODUCTION

The circadian rhythms of body functions are controlled by a central clock in the suprachiasmatic nucleus (SCN) of the hypothalamus and peripheral clocks located throughout body tissues. Light and food cues entrain these clocks to the time of day, and this synchronicity regulates a variety of physiological processes such as body temperature, sleep-wake cycles, blood pressure (BP) and glucose metabolism. Pregnancy has its cyclical characteristics and relates to a variable expression of the circadian timekeeping system (1,2). Hence, a functional circadian system is necessary during gestation (3). In mice, disruption of endogenous circadian timekeeping dramatically reduces pregnancy success (4). Circadian system alterations may also be associated with increasing risk of adverse pregnancy outcomes in humans (5,6). The sleep-wake cycle is a circadian behavioral expression that may be importantly associated with pregnancy outcomes (1). Nevertheless, few studies have explored its role in pregnancy.

Important adaptations in the metabolic system are necessary to balance fetal and placental demands with the maintenance of maternal homeostasis (2). Glucose metabolism, the main energetic substrate used by the fetus, obeys circadian oscillation, and regulates according to a large number of genes encoding glucoregulatory enzymes of rhythmic expression (7). Consequently, circadian rhythm disruption is associated with metabolic imbalance (1). Disorder of glucose metabolism is a common complication affecting 7.5% to 27.0% of pregnancies worldwide. Hyperglycemia first detected during pregnancy leads to a greater risk for adverse outcomes, notably, infants large for gestational age or macrosomia and maternal pre-eclampsia/hypertensive disorders. Disturbance in glucose metabolism is also related to preterm delivery (8). Importantly, low and middle-income patients are more affected, and this can be related to unhealthy diet and lifestyle factors (9). Therefore, events associated with maternal/neonatal risk in Gestational Diabetes Mellitus (GDM) must be identified and addressed.

Sleep-wake alterations, common during gestation (10), are related to poor pregnancy outcomes. An extensive review and meta-analysis examining sleep duration in pregnancy, involving 18,203 subjects at baseline and 1294 GDM cases with follow-up, showed that poor sleep quality and extremes of sleep duration during pregnancy are associated with the development of gestational diabetes (11).

Other sleep related variables, e.g., chronotype, may be associated with maternal and fetal outcomes in GDM patients. Chronotype is a variation in the behavior of the individual circadian rhythm that expresses different forms of synchronization of the so-called biological clock. They determine the individual’s predisposition to feel peak energy or tiredness, according to the time of day, influencing well-being and health. Morning-evening preference influences mood, eating habits, body temperature and other biological functions. For instance, evening preference has been associated with night eating and obesity (12). Evening preference is also connected with anxiety-depression and symptom severity in bipolar patients (13). In fact, the role of sleep/wake rhythm in GDM is yet to be better clarified.

The objective of this study is to evaluate morning-evening preference in patients with GDM, the association with clinical variables and maternal/neonatal outcomes.

## MATERIALS AND METHODS

### Study population and design

This was a longitudinal and prospective cohort study of GDM patients at the Center for Diabetes and Hypertension (CIDH-CE) in Fortaleza, Brazil. This is an institutional referral center unit serving approximately 600 outpatient pregnant women with diabetes per year. The study population was derived as a non-probability sampling, consecutively recruited over a 23-month period, from March 2018 to February 2020. Women with singleton pregnancy, during the second or third trimester, 20 years of age or older, diagnosed with GDM per IADPSG criteria (14) were invited to participate. The study was performed in accordance with the ethical guidelines of the 1975 Declaration of Helsinki and approved by the Ethics Committee of the Federal University of Ceará – COMEPE-UFC ethics board (2.521.562). A written informed consent was obtained from all patients after full explanation of the purpose of the study.

### Data collection

Three trained paramedical professionals obtained data using face-to-face interviews. Socio-demographics, clinical and obstetric information were collected using a structured questionnaire. Information about maternal and gestational age, parity and previous history of sleep or mood disturbances were derived from patient interviews and further confirmed through chart review. BMI and blood pressure information were verified. Objective sleep parameters such as sleep duration, efficiency and sleep mid-point were obtained through 7 days of actigraphy recording in 53 patients (Motionlogger, Ambulatory Monitoring Inc., Ardsley, NY, USA). Data regarding pregnancy and neonatal outcomes were collected in the first post-partum visit and confirmed through chart review.

### Assessments of sleep, mood and chronotype

In this work, the Horne-Östberg Morning-Evening Questionnaire (MEQ), Portuguese version (15), was used to establish the chronotype. This is a self-assessment questionnaire developed to evaluate circadian rhythm and sleep rhythm patterns. The sum of all scores in MEQ gives a result ranging from 16 to 86; scores of 41 and below indicate “evening types”, scores of 59 and above indicate “morning types”, and scores between 42-58 indicate “intermediate types”, Essentially, low scores reflect more of an evening orientation, whereas high scores reflect a morning orientation. In this study, we used a cut off of ≤ 41 to define eveningness (16). Demographic and clinical information included gestation data, comorbidities, previous history of depressive symptoms and insomnia.

Additionally, GDM patients completed a sleep log and wore an activity monitor (Motionlogger, Ambulatory Monitoring Inc., Ardsley, NY, USA) on their non-dominant wrist for five to seven consecutive 24-hour periods, including a weekend, to record activity levels at 1-minute intervals (zero crossing mode). Activity data were used to calculate (Action W-2 software; Ambulatory Monitoring) the following parameters: sleep onset time (the first of at least three consecutive minutes with an activity frequency count of 0); sleep offset time (the final activity frequency count of 0 before waking in the morning); total sleep time (TST; sleep duration minus the sum of the durations of all awakenings); sleep midpoint (the midpoint between sleep onset and sleep offset), and sleep efficiency (TST/sleep duration×100). The bedtime and waking time from each subject’s daily sleep log were used to guide the analysis of the actigraphy data recording. Additional behavioral questionnaires evaluated sleep quality (Pittsburgh Sleep Questionnaire Index – PSQI) (17), daytime sleepiness (Epworth Sleepiness Scale – ESS) (18), insomnia (Insomnia Severity Index – ISI) (19), fatigue (Fatigue Severity Scale) (20) and depressive symptoms (Edinburgh Postnatal Depression Scale) (21). All instruments were validated in the Brazilian Portuguese language and were previously used in pregnancy. Low educational level was defined if there was < 8 years of schooling. Low family income if < U$ 420.00/month and sedentary lifestyle if < 150 minutes activity per week was registered (22).

### Assessments of pregnancy outcomes

Maternal adverse outcomes included gestational weight gain, gestational age at delivery determined by the last menstrual period or ultrasound dating, Caesarian delivery, gestational hypertension and preeclampsia (23), pharmacological treatment for DM, and postpartum glucose tolerance status. Neonatal outcomes included head circumference, birth weight, Apgar score (5 and 10 minutes), being small for gestational age (SGA) defined as a birth weight < 10th percentile for gestational age by gender, or large for gestational age (LGA) defined as birth weight larger than the 90th percentile for gestational age by gender (24), macrosomia defined as birth weight greater than more than 4,000 grams (8 pounds, 13 ounces) (25), need for a neonatal intensive care unit (ICU) and prematurity defined as babies born alive before 37 weeks of pregnancy.

### Statistical methods

Data are presented as mean/standard deviation or frequency when appropriate. Fisher’s exact test examined the associations between categorical variables. A *t* test compared variables with normality and equality of variance. The Mann-Whitney U test was used for between groups comparison of behavioral scores. Factors associated with eveningness were examined using regression analyses. All variables were initially examined in univariate models. To control for confounding factors, we then performed multivariate regression analysis (Enter method) including variables that showed association in the univariate models (p < 0.2). We used a Poisson regression with robust model to calculate relative risk (RR). SPSS version 17.5.1 J software for Windows (SPSS Inc., Tokyo) was used for the above statistical analyses. A *P* value of less than 0.05 was considered a statistically significant difference.

## RESULTS

Clinical and demographic characteristics are specified in Table 1. The study involved 305 patients with GDM. Evening preference (MEQ ≤ 41) was found in 21 patients (6.9%). In detail, MEQ scores revealed that patient’s preference or chronotype were classified as morning type (N = 151; 49.5%), intermediate type (N = 133; 43.6%), and evening type (N = 21; 6.9%). In this study, evening types were compared to morning and intermediate types. As expected, actigraphic study showed a sleep delayed mid-point (p = 0.04) for the evening preference patients (Table 2). Patients with evening preference were younger, had more unstable marital status and insomnia before pregnancy. Abortion was more frequent in the older morning and intermediate types (Table 1). In addition, GDM patients with evening preference had worse sleep quality (PSQI), greater insomnia severity (ISI), more fatigue, and depressive symptoms (EDPS) (Table 2). Maternal and neonatal outcomes are described in Table 3. The presence of an evening chronotype was associated with preeclampsia [p = 0.01; OR = 0.27; CI 0.09-0.79] and a higher need for neonatal intensive care unit (NICU) treatment after birth [p = 0.02; OR = 0.23; CI .0.06-0.80]. A trend for preterm delivery was observed in GDM patients with evening chronotypes [p = 0.07; OR = 0.36; CI 0.12-1.10]. Of note, the BMI was similar among evening and non-evening preference women (Table 1), and there was no difference in the prevalence of obesity (adjusted for gestational age) among women regarding the presence or absence of pre-eclampsia (57.1% versus 44,6%; p = 0.14).

**Table 1:** Characteristics of patients with Gestational Diabetes Mellitus according to chronotype (Morning/Indifferent Intermediate Horne-Östberg MEQ ≥ 41 and Eveningness Horne-Östberg MEQ < 41)

N (%)	Total	MEQ > 41	MEQ ≤ 41	*P* value
	305	284 (93.1%)	21 (6.9%)	
**Demographics**				
Age (y)	33.0 (5.6)	33.3 (5.6)	29.9 (6.1)	(a) 0.007**
Low educational level (<8y of education)	16.3%	16.7%	15.0%	(b) 0.72
Employment	52.10%	53.50%	36%	(b) 0.07
Low family income (<U$ 420.00/month)	2.62 (2.95)	2.70 (3.06)	1.91(1.2)	(b) 0.19
Stable Marital Status	95.20%	96.50%	81%	(b) 0.01*
**Clinical Data**				
Gestational age (weeks)	29.1 (5.6)	29.2 (5.7)	28.4 (5.3)	(a) 0.53
Parity (N)	0.99 (0.98)	0.99 (0.99)	1.0 (1.0)	(a) 0.97
Abortion (N)	0.50 (1.0)	0.53 (1.0)	0.16 (0.37)	(a) 0.01*
Pre-gestational BMI	29.6 (5.5)	29.5 (5.5)	30.6 (6.4)	(a) 0.37
BMI	32.2 (5.0)	32.1 (4.9)	32.8 (6.5)	(a) 0.52
Diastolic blood pressure	73.0 (9.1)	72.7 (9.0)	75.5 (9.1)	(a) 0.18
Systolic blood pressure	115.2 (12.2)	115.0 (12.4)	116.6 (10.5)	(a) 0.58
HB1Ac	5.4 (0.5)	5.4 (0.5)	5.5 (0.4)	(a) 0.64
Fasting Blood Glucose (OGTT)	98.7 (14.8)	98.6 (14.9)	100.0 (14.8)	(a) 0.53
**Comorbidities and Risk Behavior**				
Sedentary lifestyle (<150min/activity/week)	55.1%	54.3%	66.7%	(b)0.36
Obesity	47.4%	46.4%	57.1%	(b) 0.20
Arterial hypertension	20.7%	20.2%	23.8%	(b) 0.77
Dyslipidemia	7.8%	8.2%	4.2%	(b)1.00
Depression/anxiety before pregnancy	14.0%	13.3%	23.8%	(b) 0.19
Insomnia before pregnancy	20.8%	18.3%	52.4%	(b) 0.001*

Data expressed as mean (range) or frequency (%).

PSQI: Pittsburgh Sleep Quality Index; ISI: Insomnia Severity Index; EDPS: Edinburgh Postpartum Depression Scale.

(a) *t* test

(b) or Fisher Exact test

* p < 0.05.

** p < 0.01.

**Table 2:** Behavioral Scales and Actigraphy Data in Pregnancy with Gestational Diabetes (mean ± SD; range)

Behavioral Scales	Total	MEQ > 41	MEQ ≤ 41	P values
PSQI	7.4 ± 3.6 4-17	7.2 ± 3.6 1-17	9.5 ± 4.1 3-17	0.01*
ISI	8.5 ± 5.7 0-26	8.2 ± 5.6 0-26	13.3 ± 5.4 5-25	<0.005**
EDPS	8.2 ± 5.9 0-29	7.9 ± 5.6 0-24	12.3 ± 7.7 1-29	0.009**
FSS	36.0 ± 15.9 9-74	35.1 ± 15.8 9-74	48.1 ± 12.8 20-63	<0.005**
Actigraphy Data (mean ± SE, n = 53)				
Total sleep time (TST (min)	335.2 ± 12.45	334.7 ± 12.59	341.62 ± 59.97	0.87
Sleep midpoint (h)	4:08:11 ± 0:36:35	3:43:52 ± 0:33:06	7:51:58 ± 3:27:59	0.04*
Sleep efficiency (%)	85.57 ± 1.04	85.54 ± 1.11	85.88 ± 3.35	0.92

Data expressed as mean (Range).

PSQI: Pittsburgh Sleep Quality Index; ISI: Insomnia Severity Index; EDPS: Edinburgh Postpartum Depression Scale.

Mann-Whitney test

* p < 0.05.

** p < 0.01.

**Table 3:** Pregnancy outcomes in Gestational Diabetes Mellitus according to chronotype (Morning & Intermediate – Horne-Östberg MEQ > 41 vs. Eveningness – Horne-Östberg MEQ ≤ 41)

	TOTAL	GDM MEQ > 41	GDM MEQ ≤ 41	*p* value
**Maternal Outcomes**				
Gestational weight gain	7.68 ± 6.6	7.65 ± 6.6	7.77 ± 7.2	(a) 0.94
Gestational age at delivery (weeks)	38.0 (13.9)	37.9 (2.0)	38.0 (1.6)	(a) 0.98
Cesarean delivery	84.5%	84%	88%	(b) 0.74
Gestational Hypertension	24.8%	24.3%	33.3%	(b) 0.53
Pre-Eclampsia	14.6%	13%	35.3%	(b) 0.02*
Pharmacological treatment for DM	46.8%	45.7%	61.1%	(b) 0.25
Postpartum glucose intolerance	35.7%	34.4%	57.1%	(b) 0.25
**Fetal Outcomes**				
Head circumference	34.6 (1.9)	34.5 (1.9)	35.1 (1.1)	(a) 0.35
Birth weight (g)	3279.4 (669.6)	3271.8 (681.8)	3384.2 (453.8)	(a) 0.49
APGAR Score (5’)	8.2 (1.1)	8.2 (1.2)	8.31 (0.4)	(a) 0.93
APGAR Score (10’)	9.0 (0.7)	8.98 (0.7)	9.08 (0.2)	(a) 0.68
SGA	7.8%	7.8%	6.7%	(b) 1.0
LGA	23.1%	21.9%	40%	(b) 0.21
Macrosomia	8.6%	7.7%	12.5%	(b) 0.63
Neonatal ICU	9.3%	7.8 %	26.7%	(b) 0.03*
Prematurity	13.6%	12.4%	27.8%	(b) 0.07

Data expressed as mean (Range) and frequency (%).

SGA: Small for Gestational Age; LGA: Large for Gestational Age.

(a) *t* test

(b) or Fisher Exact test

* p < 0.05

**Figure 1:**
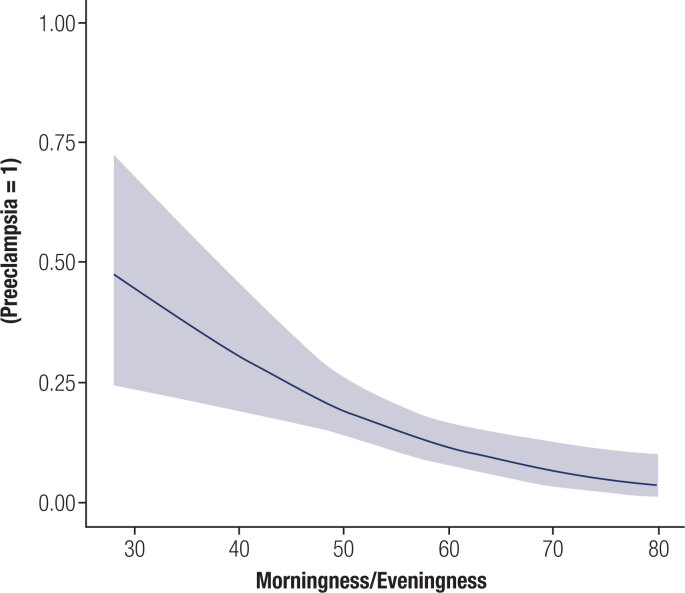
Eveningness (lower MEQ score) is associated with preeclampsia in gestational diabetes mellitus.

**Figure 2:**
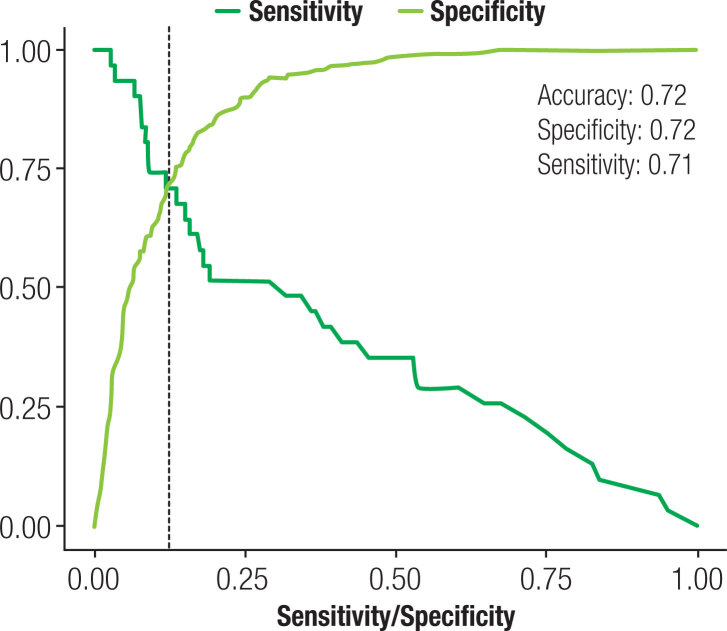
Accuracy, specificity and sensitivity of this model.

A multivariate logistic regression analysis (Poisson robust model) showed that patients with eveningness and arterial hypertension had a higher relative risk for pre-eclampsia, and these results were maintained after controlling for age, poor sleep quality, fatigue and depressive symptoms: eveningness [p < 0.005; RR = 0.94; CI: 0.91-0.98] and hypertension [p < 0.005; RR = 4.6; CI: 2.48-8.54] (Table 4). A linear regression analysis confirmed that lower MEQ scores were associated with preeclampsia [p = 0.002; 0.94; CI 0.90-0.97] (Figure 1). The accuracy, specificity and sensitivity of this model are presented in Figure 2.

**Table 4:** Logistic regression multivariate analysis (using Poisson robust model) of factors influencing pre-eclampsia before and after controlling for variables (p < 0.2)

Variables	Pre-eclampsia	
Arterial Hypertension	5.3 [2.95-9.39]	<0.005**
Age	0.97 [0.92-1,03]	0.36
MEQ	0.94 [0.90-0.97]	.002*
PSQI	0.91 [0.81-1.02]	0.11
EDPS	0.94 [0.88-1.01]	0.10
FSS	0.98 [0.95-1.00]	0.11
Controlled for Age, Body Mass Index, Arterial Hypertension, PSQI, EDPS, FSS		
MEQ	0.94 [0.91- 0.98]	<0.005**
Arterial Hypertension	4.6[2.48-8.54]	<0.005**
PSQI	0.95[0.86-1.05]	0.27
EDPS	0.94 [0.88-1.00]	0.05
FSS	0.98 [0.96-1.00]	0.16
Age	0.98[0.93-1.03	0.48

PSQI: Pittsburgh Sleep Quality Index; EDPS: Edinburgh Postnatal Depression Scale; MEQ: Morningness Eveningness Questionnaire; FSS: Fatigue Severity Scale.

** p < 0.005

## DISCUSSION

This study is the first to evaluate sleep alterations and the chronotype in a significant group of women with GDM. The present data confirm that sleep alterations are common in GDM. Moreover, patients with evening preference had worse sleep quality, increased severity of insomnia, depressive symptoms, and fatigue. Importantly, eveningness was associated with the development of preeclampsia and a greater need for NICU. These findings indicate that the evening chronotype has an unfavorable association with maternal and neonatal outcomes.

In this work, the evening chronotype was found in 6.9% of patients, a small percentage of the study population. Eveningness is generally observed in approximately one fifth to one third of the population in Brazil and is naturally more expressed in young individuals (26). Lower levels of evening chronotype have also been reported in other adult population studies (Korea: 5.9%; New Zealand: 5.6%) (27,28). Research indicates that pregnancy induces an earlier chronotype in both mice and women (29) and this might contribute to the low prevalence of eveningness presently found. In partial agreement with our findings, a report from Finland evaluating the chronotype of 1,653 normal pregnant women using a shortened version of the morningness-eveningness questionnaire, described eveningness in about 13% of patients (30).

The present study was conducted in Northeastern Brazil, which has a latitudinal cline favoring generous and stable sunlight exposure throughout the year. Sunlight exposure is considered a strong *zeitgeber* that entrains circadian rhythms. However, it must be kept in mind that multiple *zeitgebers* interact to synchronize the circadian rhythms. Despite the influence of daylight exposure, artificial light, social interaction, physical exercise, and feeding patterns are also implicated as important time cues (31). Ontogenetic characteristics of chronotype have formerly been recognized. For instance, younger individuals have a clear tendency toward eveningness, whereas older age is strongly associated with morningness (31). In fact, chronotype has been accepted to be maintained until the age of 35: from this age and, thereafter, morningness increases. Evidence indicates that females reach the maximum of their eveningness sooner than males and this gender effect disappears around the age of 50, the average age of menopause (32). In accordance with this concept, a younger age was also found among pregnant women with evening preference in this group. Studies involving larger samples may further clarify differences in circadian behavior regarding pregnancy.

Previously, we compared the circadian behavior of GDM patients with 93 non-GDM pregnant women with similar gestational age (control group). The GDM group was older and more obese. Overall, a higher prevalence of morning preference in both groups was observed (52%), with a non-significant difference in evening preference rates: (GDM 6.1% vs. non-GDM 3.3%, *P* = 0.2). Interestingly, a linear analysis of MEQ showed a higher score in non-GDM patients, suggesting a trend toward a morning preference in the control group (*P* = 0.05).

Evening chronotype, independent of the presence of diabetes or other comorbidities, has been associated with a greater risk of sleep alterations and mood symptoms [13]. In a previous report on non-diabetic pregnancy, evening chronotype was not related to adverse outcomes nor gestational diabetes (33). Conversely, another study retrospectively evaluating 313 pregnant women showed that lower MEQ was associated with preterm delivery and preeclampsia (34). Furthermore, evening chronotype has also been related to sleep problems and unhealthy life habits during pregnancy (30). Importantly, in our analysis, GDM patients with evening chronotype had a history of insomnia before pregnancy and more unstable marital status. It could be hypothesized that eveningness was present before pregnancy.

In partial agreement with our findings, a previous study observed an association between circadian rhythm disorders and pre-eclampsia in shift workers (35). A key strength of our study is that it is the first to report an association of preeclampsia and evening chronotype in GDM pregnancy. A connection exists between sleep disorders, circadian rhythm dysfunction, insulin resistance and arterial hypertension. The interrelationships between hypertensive syndromes during pregnancy and rhythm disorders have aroused much interest from the scientific community. An extensive study showed that arterial hypertension was related to long and short sleep duration (36). Interestingly, in the present work, arterial hypertension and MEQ scores were the only independent measures associated with preeclampsia.

Blood pressure varies over a 24-hour period following a circadian rhythm profile. Healthy individuals experience a 10%-20% decrease in BP at night (37). The loss of this rhythm has been shown to be an initial event responsible for cardiovascular complications both inside and outside pregnancy, and the suppression of a nighttime pressure drop is a hallmark of eclamptic syndromes in pregnancy (38,39). Therefore, the hypothesis that the misalignment of circadian rhythm would be a potential risk factor for pre-eclampsia is intriguing and could offer a new field of investigation and approach to this disease related to adverse pregnancy outcomes.

**Figure 3:**
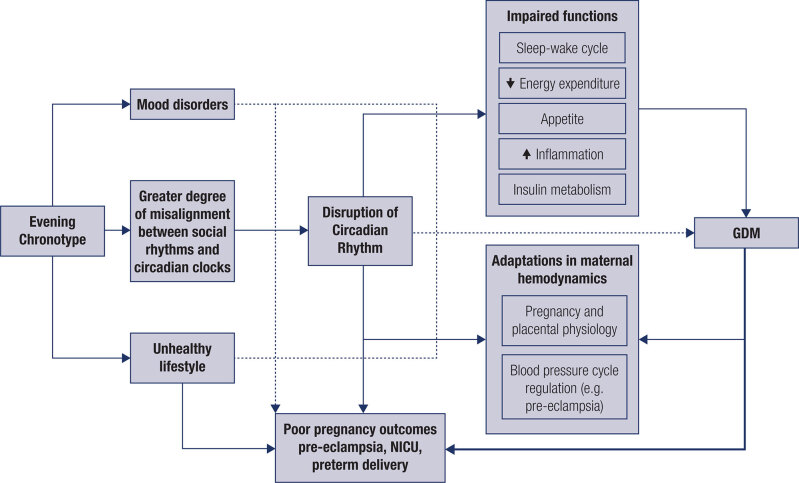
A theoretical model: The relationship among evening chronotype, gestational diabetes, other key variables and outcomes.

Depressive symptoms are common and a genuine concern in GDM (40). Sleep disorders are also widely recognized as having an adverse effect on glucose metabolism and as a risk factor for the development of complications in pregnancy, including GDM (41). Our study confirms a relationship of evening chronotype with sleep and mood disorders similar to previous reports on other clinical conditions (42). Importantly, chronotype influences neuroendocrine secretion, alertness, cognition, feeding, renal, ovary and pulmonary function, and many of these functions are connected to glucose control and other metabolic dysfunction (43). In this study, depressive symptoms were not associated with hypertensive syndromes in pregnancy or a greater need for NICU admission. A suggestion on how chronotype, gestational diabetes, other key variables and outcomes might be related is illustrated in a theoretical model (Figure 3).

Limitations to this study must be acknowledged. A reduced number of GDM patients with evening preference undermined the power of the analysis. Possibly, an explanation for these findings is the trend for morning preference in pregnancy. Actigraphy data confirmed a delayed sleep mid-term point in GDM patients with evening preference. Furthermore, MEQ scores were independently associated with pre-eclampsia.

All these findings indicate a negative influence of evening chronotype in maternal-neonatal outcomes e.g., preeclampsia and a greater need for NICU admission in GDM. Eveningness was related to poor sleep quality, higher severity of insomnia, depressive symptoms and fatigue. Importantly, chronotype can be established with simple and affordable tasks during routine prenatal care and could provide a feasible way in the prediction of adverse pregnancy outcomes. Attention to healthy habits including morning exposure to bright light, exercise and a reduction of screen blue light at night may improve the care of GDM patients with evening preference.
